# Supplementary material for: Pyruvate Dehydrogenase Kinase 1 inhibition mediated oxidative phosphorylation enhancement in cartilage promotes osteoarthritis progression
Source: BMC Musculoskelet Disord. 2023 Jul 20;24:597. doi: 10.1186/s12891-023-06585-6 (PMC10357736; doi:10.1186/s12891-023-06585-6)
Supplement: Supplementary file 1 — Additional file 1. [file 12891_2023_6585_MOESM1_ESM.zip › BP.Gsea.1653623667859/GOBP_CELLULAR_METABOLIC_COMPOUND_SALVAGE.html]

Details for gene set GOBP\_CELLULAR\_METABOLIC\_COMPOUND\_SALVAGE[GSEA]

|  || Dataset | 55457\_collapsed\_to\_symbols.cls.cls #OA\_versus\_normal.cls.cls #OA\_versus\_normal\_repos |
| Phenotype | cls.cls#OA\_versus\_normal\_repos |
| Upregulated in class | OA |
| GeneSet | GOBP\_CELLULAR\_METABOLIC\_COMPOUND\_SALVAGE |
| Enrichment Score (ES) | 0.4401373 |
| Normalized Enrichment Score (NES) | 1.4816551 |
| Nominal p-value | 0.0389016 |
| FDR q-value | 0.38697776 |
| FWER p-Value | 1.0 |
Table: GSEA Results Summary

  

Fig 1: Enrichment plot: GOBP\_CELLULAR\_METABOLIC\_COMPOUND\_SALVAGE      
 Profile of the Running ES Score & Positions of GeneSet Members on the Rank Ordered List

  

| SYMBOL | TITLE | RANK IN GENE LIST | RANK METRIC SCORE | RUNNING ES | CORE ENRICHMENT || 1 | DGUOK | deoxyguanosine kinase [Source:HGNC Symbol;Acc:HGNC:2858] | 96 | 0.917 | 0.1129 | Yes |
| 2 | ADK | adenosine kinase [Source:HGNC Symbol;Acc:HGNC:257] | 127 | 0.878 | 0.2258 | Yes |
| 3 | APIP | APAF1 interacting protein [Source:HGNC Symbol;Acc:HGNC:17581] | 643 | 0.607 | 0.2656 | Yes |
| 4 | DCK | deoxycytidine kinase [Source:HGNC Symbol;Acc:HGNC:2704] | 920 | 0.537 | 0.3146 | Yes |
| 5 | APRT | adenine phosphoribosyltransferase [Source:HGNC Symbol;Acc:HGNC:626] | 1178 | 0.481 | 0.3577 | Yes |
| 6 | AMPD1 | adenosine monophosphate deaminase 1 [Source:HGNC Symbol;Acc:HGNC:468] | 1789 | 0.385 | 0.3609 | Yes |
| 7 | PDXK | pyridoxal kinase [Source:HGNC Symbol;Acc:HGNC:8819] | 1923 | 0.370 | 0.3991 | Yes |
| 8 | ADI1 | acireductone dioxygenase 1 [Source:HGNC Symbol;Acc:HGNC:30576] | 2004 | 0.360 | 0.4401 | Yes |
| 9 | MTAP | methylthioadenosine phosphorylase [Source:HGNC Symbol;Acc:HGNC:7413] | 3324 | 0.219 | 0.3664 | No |
| 10 | AMPD3 | adenosine monophosphate deaminase 3 [Source:HGNC Symbol;Acc:HGNC:470] | 4174 | 0.140 | 0.3189 | No |
| 11 | ENOPH1 | enolase-phosphatase 1 [Source:HGNC Symbol;Acc:HGNC:24599] | 5615 | 0.023 | 0.2101 | No |
| 12 | ADSL | adenylosuccinate lyase [Source:HGNC Symbol;Acc:HGNC:291] | 5685 | 0.017 | 0.2069 | No |
| 13 | BHMT2 | betaine--homocysteine S-methyltransferase 2 [Source:HGNC Symbol;Acc:HGNC:1048] | 6165 | -0.018 | 0.1721 | No |
| 14 | HPRT1 | hypoxanthine phosphoribosyltransferase 1 [Source:HGNC Symbol;Acc:HGNC:5157] | 6954 | -0.076 | 0.1209 | No |
| 15 | BHMT | betaine--homocysteine S-methyltransferase [Source:HGNC Symbol;Acc:HGNC:1047] | 7250 | -0.099 | 0.1110 | No |
| 16 | TK2 | thymidine kinase 2 [Source:HGNC Symbol;Acc:HGNC:11831] | 7468 | -0.116 | 0.1093 | No |
| 17 | ADA | adenosine deaminase [Source:HGNC Symbol;Acc:HGNC:186] | 8105 | -0.163 | 0.0814 | No |
| 18 | CDA | cytidine deaminase [Source:HGNC Symbol;Acc:HGNC:1712] | 8324 | -0.180 | 0.0881 | No |
| 19 | AMPD2 | adenosine monophosphate deaminase 2 [Source:HGNC Symbol;Acc:HGNC:469] | 9530 | -0.276 | 0.0308 | No |
| 20 | PNP | purine nucleoside phosphorylase [Source:HGNC Symbol;Acc:HGNC:7892] | 9657 | -0.288 | 0.0588 | No |
| 21 | UCK2 | uridine-cytidine kinase 2 [Source:HGNC Symbol;Acc:HGNC:12562] | 10481 | -0.371 | 0.0436 | No |
| 22 | UCKL1 | uridine-cytidine kinase 1 like 1 [Source:HGNC Symbol;Acc:HGNC:15938] | 11515 | -0.503 | 0.0293 | No |
| 23 | UPP1 | uridine phosphorylase 1 [Source:HGNC Symbol;Acc:HGNC:12576] | 12013 | -0.595 | 0.0689 | No |
Table: GSEA details [plain text format]

  

Fig 2: GOBP\_CELLULAR\_METABOLIC\_COMPOUND\_SALVAGE      
 Blue-Pink O' Gram in the Space of the Analyzed GeneSet

  

Fig 3: GOBP\_CELLULAR\_METABOLIC\_COMPOUND\_SALVAGE: Random ES distribution      
 Gene set null distribution of ES for **GOBP\_CELLULAR\_METABOLIC\_COMPOUND\_SALVAGE**
